# A wearable tool for real-time dose monitoring during cancer radiation therapies

**DOI:** 10.1126/sciadv.adt7633

**Published:** 2025-04-25

**Authors:** Ilaria Fratelli, Sara M. Carturan, Francesco Tommasino, Laura Basiricò, Felix Pino, Antonio Valletta, Marcello Campajola, Matteo Rapisarda, Sabrina Calvi, Mattia Scagliotti, Andrea Ciavatti, Luca Tortora, Enrico Verroi, Jessica C. Delgado, Lorenzo Margotti, Camilla Bordoni, Giulia Napolitano, Sandra Moretto, Alberto Aloisio, Ettore Sarnelli, Paolo Branchini, Luigi Mariucci, Alberto Quaranta, Beatrice Fraboni

**Affiliations:** ^1^ Department of Physics and Astronomy, University of Bologna, Viale Berti Pichat 6/2, 40127 Bologna, Italy.; ^2^ INFN, Sezione di Bologna, Viale Berti Pichat 6/2, 40127 Bologna, Italy.; ^3^ Department of Physics and Astronomy, University of Padova, Via Marzolo 8, Padova, Italy.; ^4^ INFN-Laboratori Nazionali di Legnaro, Viale dell’Università 2, Legnaro, Italy.; ^5^ Department of Physics, University of Trento, Via Sommarive 9, Povo, 38123 Trento, Italy.; ^6^ Trento Institute for Fundamental Physics and Applications, Via Sommarive 9, Povo, 38123 Trento, Italy.; ^7^ INFN, Sezione di RomaTre, Via Della Vasca Navale 84, 00146 Roma, Italy.; ^8^ Institute for Microelectronics and Microsystems, IMM-CNR, Via Del Fosso Del Cavaliere, 100, 00133 Roma, Italy.; ^9^ INFN-Sezione di Napoli-Complesso Univ. di Monte S. Angelo, Edificio G, Via Cintia, 80126 Napoli, Italy.; ^10^ Department of Physics, Complesso Univ. di Monte S. Angelo, University of Naples Federico II, Edificio G, Via Cintia, 80126 Napoli, Italy.; ^11^ Department of Physics, University of Rome Tor Vergata, Via della Ricerca Scientifica 1, 00133 Rome, Italy.; ^12^ Department of Sciences, Roma Tre University, Via della vasca navale 84, 00146 Roma, Italy.; ^13^ INFN, Sezione di Padova, Via Marzolo 8, Padova, Italy.; ^14^ CNR-SPIN, Via Campi Flegrei, 34, 80078 Pozzuoli, NA, Italy.; ^15^ Task Force di Bioelettronica, University of Naples Federico II, Napoli, Italy.; ^16^ Department of Industrial Engineering, University of Trento, Via Sommarive 9, Povo, 38123 Trento, Italy.

## Abstract

We report on a wearable, human tissue-equivalent, real-time dosimeter designed to quantitatively monitor radiation absorbed by patients during cancer treatments. The fully organic device has been characterized under actual clinical conditions using a high-energy proton beam and an anthropomorphic phantom, with the aim to simulate a prostate cancer proton therapy treatment. We achieved a full control over the dosimeter operation, and we verified its linear response with the received dose. We demonstrate that, by a proper functionalization of the polysiloxane-based scintillator, it is possible to target the effective detection of different kinds of ionizing radiation. Specifically, besides protons, we develop a device able to detect thermal neutrons, targeting its use during Boron Neutron Capture Therapy. This work demonstrates how organic indirect detectors can be considered a universal radiation detecting platform able to monitor in real time and in situ the dose absorbed by patients during cancer treatments under different kinds of radiation.

## Teaser

A fully organic, wearable, tissue-equivalent, real-time dosimeter to monitor dose during cancer therapy is reported.

## INTRODUCTION

Cancer is a major cause of global mortality, accounting for approximately one in six deaths. In 2022, the World Health Organization reported 20 million new cases of cancer and 9.7 million deaths from cancer worldwide. The burden of cancer is projected to increase by ~77% by 2050, placing an even greater burden on health systems, individuals, and communities (*1*). One of the most common approaches for treating patients with cancer is radiotherapy.

In radiotherapy, high-energy ionizing radiation is used to kill cancer cells, disrupting or limiting their growth. The most modern treatment plans foresee the usage of linear accelerators to generate beams of electrons or x-rays in the megavolt range, radioisotopes that produce gamma rays, cyclotrons to produce high-energy protons, and reactors for thermal neutrons. Depending on the ionizing radiation used, it is possible to exploit different mechanisms of radiation-matter interaction leading to a different release of energy within the patient body. One of the biggest challenges in radiotherapy practice is to deliver the radiation selectively to the regions where the malignant cells are located because ionizing radiation can injure healthy tissues surrounding the target volumes. Despite many techniques have been implemented to maximize the dose released in the target while minimizing that in healthy tissues (*2*, *3*), toxicities may still occur due to the mispositioning of the patient because minor movements (e.g., breathing) can alter the targeted volume of the organ and the received dose, thus affecting the effectiveness of the treatment. Therefore, an experimental real-time, in situ dose monitoring during therapy using a thin, wearable, low-interfering, and portable device would be a game changer, notably improving the quality and precision of care of patients with cancer.

In the past years, detectors based on organic semiconductors proved to be an excellent candidate for the development of a new generation of personal dosimeters. They offer several and unique advantages such as (i) the possibility to be deposited from solution by low-cost and low-temperature printing techniques envisaging for flexible and easy-scalable devices onto curved and large surfaces; (ii) their density and chemical composition make them human tissue equivalent in terms of radiation absorption avoiding for postprocess and complex calibration procedures. For this reason, in past last years, several examples of direct and indirect organic-based detectors have been reported in the literature, also assessing their characterization under actual clinical conditions (*4*–*20*).

We recently reported on the characterization under 5-MeV (mega–electron volt) proton beams (*21*) of fully organic and flexible indirect detectors, where a polysiloxane-based scintillator and an organic phototransistor (OPT) were coupled, providing an optimal mechanical and optical coupling, granting a conformable integrated device. Thanks to this preliminary characterization, we further investigated the performance and operating principles of such technology and we developed an analytic model to reproduce the detector’s response and provide a full control on the dosimetric characterization under actual clinical conditions. In this work, we present the full characterization of this fully organic detecting system in a medical environment, simulating the real-time and in situ monitoring of the dose typically absorbed by a patient during radiation treatments. First, we report the characterization under 200-MeV proton beams typically used for prostate cancer irradiation therapy. Second, we report the preliminary test of the device customized for the detection of thermal neutrons used during Boron Neutron Capture Therapy (BNCT) by coupling the OPT with a specifically developed organic scintillator. BNCT is a thermal neutron-based therapy at the cutting edge for the selective treatment of certain kinds of cancers (head and neck tumors), which relies on the killing effect produced by the selective concentration of ^10^B atoms in tumor cells and their subsequent thermal neutron irradiation. The growing interest in this new therapy is demonstrated by the increasing of clinical trials and by the construction of accelerator-based BNCT facilities, which will substitute the nuclear reactors.

The aim of this work is to demonstrate that, thanks to a proper functionalization of the scintillating material, the here reported full-organic indirect detectors represent a universal platform for the development of a wearable, conformable, and portable personal dosimeters to be used for in situ and real-time monitoring of the absorbed dose during different cancer radiation treatments.

## RESULTS

**Fig. 1.**
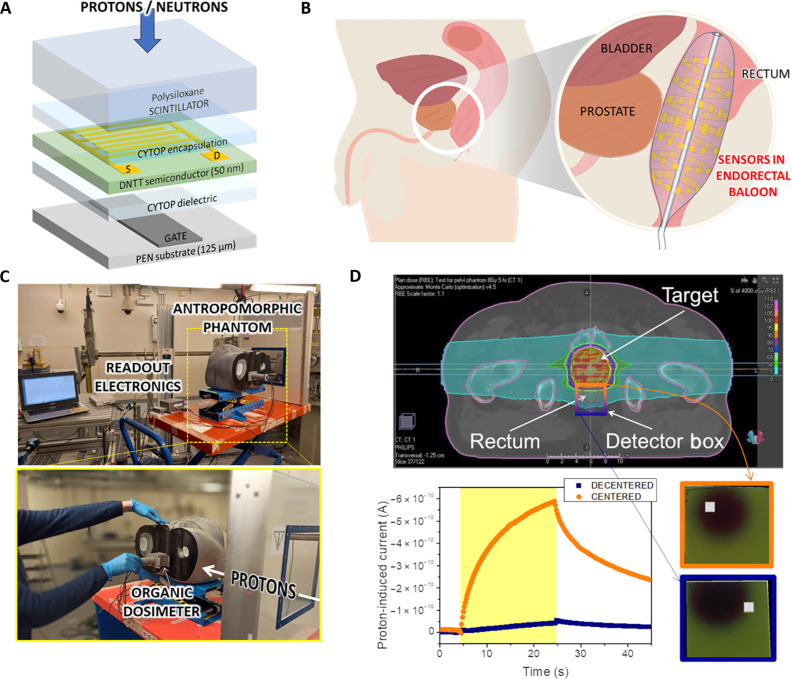
Fully organic indirect detector for the real-time dose monitoring during radiation therapies. (**A**) Schematics of the detector’s architecture. The layer thicknesses are out of scale. (**B**) Rendering of the endorectal balloon used during the treatment. The inner walls of the balloon are covered with a multipixel matrix formed by our detectors. (**C**) Pictures of the experimental setup. The irradiation tests have been performed in the experimental room of the Trento Proton Therapy Center (TIFPA). Our detector has been placed inside an anthropomorphic phantom in different positions about the prostate. (**D**) (Top) Representative treatment plan for a patient with prostate cancer treated with two opposing proton fields, as obtained by means of the TPS Raystation (Raysearch Laboratories) with the Monte Carlo dose computation engine. The proton beam energy was in the range (162 to 197 MeV). The colored box is centered at the rectum position. The orange part is close to the prostate and centered inside the primary beam spot, whereas the blue part is at the end of the range of the primary beam. The two insets show two radiochromic sheets reporting the beam shapes and the position of our detector when it was centered (orange) and decentered (blue) from the proton beam. (Bottom) Proton-induced current by the proton beam collected with the centered (orange) and decentered (blue) detector.

The schematic of the fully organic indirect detector is reported in Fig. 1A. The device is formed by an OPT coupled with a polysiloxane-based scintillator. The OPT’s architecture and fabrication process has been already described in the literature (*22*). The device has been realized onto a polyethylene naphthalate (PEN) substrate (thickness = 100 μm), and it is based on a dinaphtho[2,3-*b*:2′,3′-*f*]thieno[3,2-*b*]thiophene (DNTT) semiconducting layer. A 500-μm-thick polysiloxane-based scintillator has been coupled on the top of the photodetector using an optical resin, which minimizes the light losses and preserves the mechanical flexibility of the coupled device (*23*). Here, depending on the radiation to be detected, the scintillator can be functionalized to target and maximize the specific radiation-matter interaction. For instance, for the test under protons, we used two different scintillators, namely, polymethylphenylsiloxane (PSS100) and polyvinylphenyl-*co*-phenylmethyl siloxane (PVP-MPS) (*21*). Instead, for the test under thermal neutrons we used a different polysiloxane scintillator (DMSV21 polymeric matrix) functionalized with an inorganic part: ZnS:Ag (EJ600) and ^6^Li_2_^10^B_4_O_7_ nanoparticles, which are capable to interact with thermal neutrons through some nuclear reactions [i.e., n + ^6^Li → ^3^H (2.73 MeV) + ^4^He (2.05 MeV); n + ^10^B → ^7^Li + ^4^He (1.47 MeV) + γ; n + ^10^B → ^7^Li + ^4^He (1.78 MeV)]. The coupling between the flexible OPT and the appropriate polysiloxane-based scintillator gives rise to a comprehensive detection platform encompassing different radiations in accordance with the scintillator functionalization used.

We assess the mechanical flexibility of the device by testing the proton detecting performances when it was kept bent down to a curvature radius of 5 mm, and we also provide the proof of the scalability of the technology onto large areas (fig. S1) (*21*). A unique property offered by a full-organic detector is the human tissue equivalence. The chemical composition and density of organic materials forming the device mimic the human body in terms of energy absorption, envisaging the possibility to avoid complex calibration procedures in its actual employment as a personal dosimeter. In table S1 (Supplementary Materials), we report the relevant features in terms of biological equivalence (i.e., effective atomic number *Z_eff_* and electronic density) of the siloxane-based scintillators calculated using the equation reported by Murty (*24*) and compared to those reported for human tissues and water. In addition, thanks to the adopted thin film configuration, the device proves to be a low-interfering tool that can be placed in situ, between the target and the radiation source, without perturbing the radiation field and without interfering with the treatment plan. Figure 1B shows a possible employment of this technology: A large multipixel device is placed on the wall of an endorectal balloon used during prostate cancer treatment to stabilize the organ during irradiation and to push the surrounding organs far away from the target of the primary beam. In this application, the monitoring of the radiation dose is crucial to verify the correct delivery of energy and, if necessary, to correct any misalignment issues in real time. In the specific case reported here, by placing the dosimeter inside the endorectal balloon, we are able to spatially monitor the dose delivered next to the prostate and at the rectum position to assure the effectiveness of the therapy and to prevent side effects due to the irradiation of the surrounding healthy tissues.

To simulate and mimic actual clinical conditions, we used a 3D-printed anthropomorphic phantom reported in Fig. 1C (for technical details, see the Materials and Methods and fig. S2). The dosimeter was placed in a dedicated sample holder that mimics the endorectal balloon and that allows to test different positions within the phantom pelvis. Figure 1D reports a representative treatment plan for a patient with prostate cancer treated with two opposing proton fields, as obtained by means of the TPS Raystation (Raysearch Laboratories) with the Monte Carlo dose computation engine. The proton beam energy was in the range (162 to 197 MeV). The color scale indicates the biological dose [Relative Biological Effectiveness (RBE) = 1.1]. During the irradiation, carried out at the experimental room of the Trento Proton Therapy Center (TIFPA), the dosimeter has been tested in two different positions: close to the target of the treatment (i.e., prostate) and far from it (i.e., rectum). The detector response (i.e., the electrical output current induced by the radiation, monitored in real time) collected when it is centered/decentered with respect to the proton beam is clearly distinguishable.

**Fig. 2.**
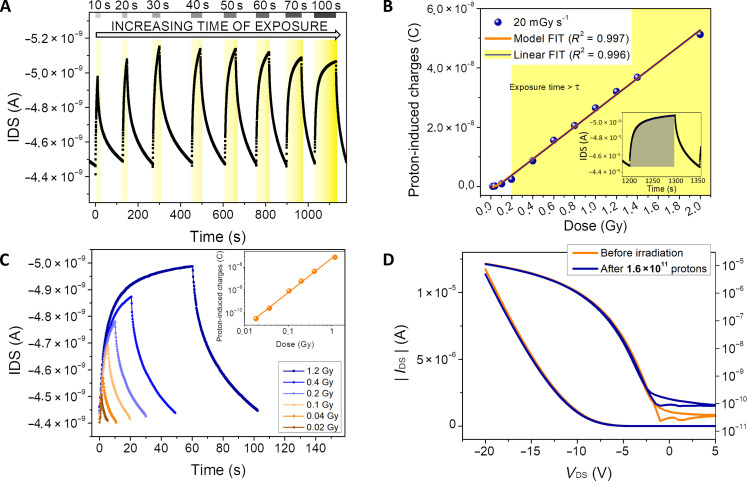
Dosimeter characterization under a 200-MeV proton beam. (**A**) Dynamic response of the detector with a PSS100-based scintillator for increasing total doses of proton irradiations (time of exposure in the range [10 to 100] s, 20 mGy s^−1^, total dose range [0.2 to 2] Gy). The yellow shadows indicate the irradiation time windows. OPT working bias voltages: *V*_DS_ = −1 V, *V*_GS_ = −4 V. (**B**) Proton-induced charges at different total doses. The proton-induced charges are calculated as the integral of the dynamic curve reported in the inset and highlighted in blue. Experimental data are fitted by our model (*21*) (orange line) from which the characteristic time of the sensor (τ = 11 ± 1 s) can be extracted. For exposure times larger than this characteristic time of the detector (i.e., doses > 220 mGy), this trend line can be approximated to a linear curve (blue line), demonstrating the dose linearity. (**C**) Dynamic response of the detector at low doses (time of exposure in the range [1 to 60] s, 20 mGy s^−1^, total dose range [0.02 to 1] Gy). The lowest detected dose is 20 mGy. The inset shows the proton-induced charges as a function of the total dose. (**D**) Transfer characteristics of the OPT before and after irradiation at 1.6 × 10^11^ total protons (15.1 Gy) for an exposure time of *t*_exp_ = 30 min. Forward and reverse curves are reported.

The proton beam is attenuated by the human tissues (i.e., 15.5 cm) before impinging onto the device, losing 82 MeV of the primary energy. The setup configuration and the SRIM simulation [Stopping and Range of Ions in Matter Monte Carlo code (*25*)] of the energy released inside the phantom are reported in the Supplementary Materials (fig. S2). Figure 2 shows the full dosimetric characterization of the device. When the protons impinge onto the detector, they release energy by ionization in the 500-μm–thick scintillator that emits light centered at 430 nm, which is then detected by the OPT. The proper match between the scintillator emission spectrum and the DNTT absorption one has already been reported in our previous work (*21*). Figure 2A shows the electrical output signals induced by 200-MeV proton irradiation cycles varying the exposure time in the range [10 to 100] s. The proton beam has been calibrated using a commercial dosimeter (MARKUS), and the dose rate was set at 20 milligray (mGy) s^−1^ (i.e., 5 × 10^8^ H^+^ s^−1^). During the irradiation, the OPT was electrically connected, biased in dc at low voltages (*V*_DS_ = −1 V, *V*_GS_ = −4 V; see fig. S3) and the *I*_DS_ current was monitored in real time using a benchtop source meter. The detector was subjected to an initial conditioning (i.e., biasing in the dark condition) for 3 min to reach the dynamic equilibrium of the system and reduce bias stress effects to negligible values. The radiation extra-charges induced by different total amounts of absorbed dose are calculated by integrating the current peak during the irradiation, as highlighted in the inset in Fig. 2B (blue shadow).

In agreement with preliminary results of this device exposed to 5-MeV protons (*21*), where we developed an analytic model to reproduce the device response, we have here used the model under actual clinical conditions and under 200-MeV proton beams to reach the full control on the dosimeter operation. We were able to identify the dose range in which the device shows a linear response and in which it can be thus reliably used as a dosimeter during clinical treatment, as described hereafter.

We considered the kinetic equation (Eq. 1) describing the dynamic of the electrical current following an irradiation starting at time *t*_0_

$$I_{\text{DS}}\left(t\right)=\frac{a}{b}-\frac{a}{b}e^{-b\left(t-t_{0}\right)},\text{with}\;I_{\text{DS}}\left(t_{0}\right)=0$$ (1)

where *b* is defined as *b* = 1/τ (τ is the characteristic relaxation time of the traps induced by radiation exposure) and *a* depends linearly on the dose rate and on the density of centers that can host a trap. The dynamics predicted by Eq. 1 is a linear increase in the photocurrent for *t* < τ and a saturation for *t* > τ, in agreement with the experimental measurements reported in Fig. 2 (B and C).

The charges induced by protons are obtained as the integral of the current

$$\begin{array}{c} \int_{t_{0}}^{t_{1}}I_{\text{DS}}\left(t\right)dt=\int_{t_{0}}^{t_{1}}\left(\frac{a}{b}-\frac{a}{b}e^{-b\left(t-t_{0}\right)}\right)dt=\frac{a}{b}∆t+\frac{a}{b^{2}}e^{-b∆t}\\ +c\;\text{with}\;∆t=t_{1}-t_{0}\;\text{and}\;c=-\frac{a}{b^{2}} \end{array}$$ (2)

When the exposure time Δ*t* >> τ (i.e., *b*Δ*t* >> 1), $e^{-b\Delta t}$ is negligible and the integral of the current results linearly dependent on the exposure time (i.e., total dose absorbed), whereas for short exposure times (i.e., Δ*t* << τ), the predicted increase in this quantity is superlinearly dependent on Δ*t*, as can be readily verified by a Taylor series expansion of the exponential.

By fitting the curve reported in Fig. 2B, for a dose rate of 20 mGy s^−1^, we obtained *a* = (5.2 ± 0.6) × 10^−11^ C s^−2^; *b* = (0.09 ± 0.01) × s^−1^ (τ = 11 ± 1 s); and *c* = (−6.0 ± 0.8) × 10^−9^ C (in good agreement with the theoretical value $-\frac{a}{b^{2}}$). This calculation demonstrates that, for exposure times *t* > τ (i.e., doses > 220 mGy), the dosimeter provides an excellent linear response with increasing dose, as reported in Fig. 2B, and that it can be reliably used to monitor in real time the dose delivered to the patient during radiation treatments. From the linear fit (blue line), we extract the sensitivity of the detector as the slope of the fitting curve, which results in *S* = (2.72 ± 0.06) × 10^−8^ C Gy^−1^. By normalizing the sensitivity for the detector active area (i.e., 0.08 cm^2^), the detector provides *S*_A_ = (3.40 ± 0.07) × 10^−7^ C Gy^−1^ cm^−2^. This value of sensitivity is more than one order of magnitude higher than the one reported for commercial proton dosimeters such as ionization chambers (PTW Advanced Markus Chamber, *S*_A_ = 0.8 × 10^−9^ C Gy^−1^ cm^−2^) and diamond solid state detector (PTW microDiamond, S_A_ = 6.7 × 10^−9^ C Gy^−1^ cm^−2^). In addition, the detector showed a reproducible, repeatable, and stable response as demonstrated in the Supplementary Materials (see figs. S4 and S5), showing how the calibration curve remains unchanged by increasing and decreasing the total dose and after 24 hours of operation.

The here discussed devices could be further exploited to evaluate the side effects of the therapy by monitoring eventual misalignments and unwanted irradiation of surrounding healthy tissues. To assess such functionality, we irradiated the device lowering the exposure time (i.e., the total dose absorbed). The detector demonstrates to provide a clear signal down to 1 s of exposure (i.e., 20 mGy) (Fig. 2C) due to the generation of extra-charges upon the release of energy by impinging protons. As discussed before, in the range [20 to 220] mGy, the detector does not provide a linear response (see the log-log plot in the inset in Fig. 2C).

We also assessed the radiation hardness of the dosimeter by exposing it to 1.6 × 10^11^ total protons (15.1 Gy) for a total time of 30 min. In Fig. 2D, the transfer characteristics in the linear regime (*V*_DS_ = −1 V) of the OPT are reported before and after the irradiation. The two curves perfectly overlap, indicating the very good radiation hardness of the dosimeter. The only slight difference is a current increase in the off state, accountable by the kinetic model developed in our previous work (*21*) due to the long recovery time of the OPT response to ultraviolet-visible light emitted by the scintillator. Further details on the photocurrent dynamics and recovery during and after radiation exposure and their interpretation can be found in our previous work (*21*).

**Fig. 3.**
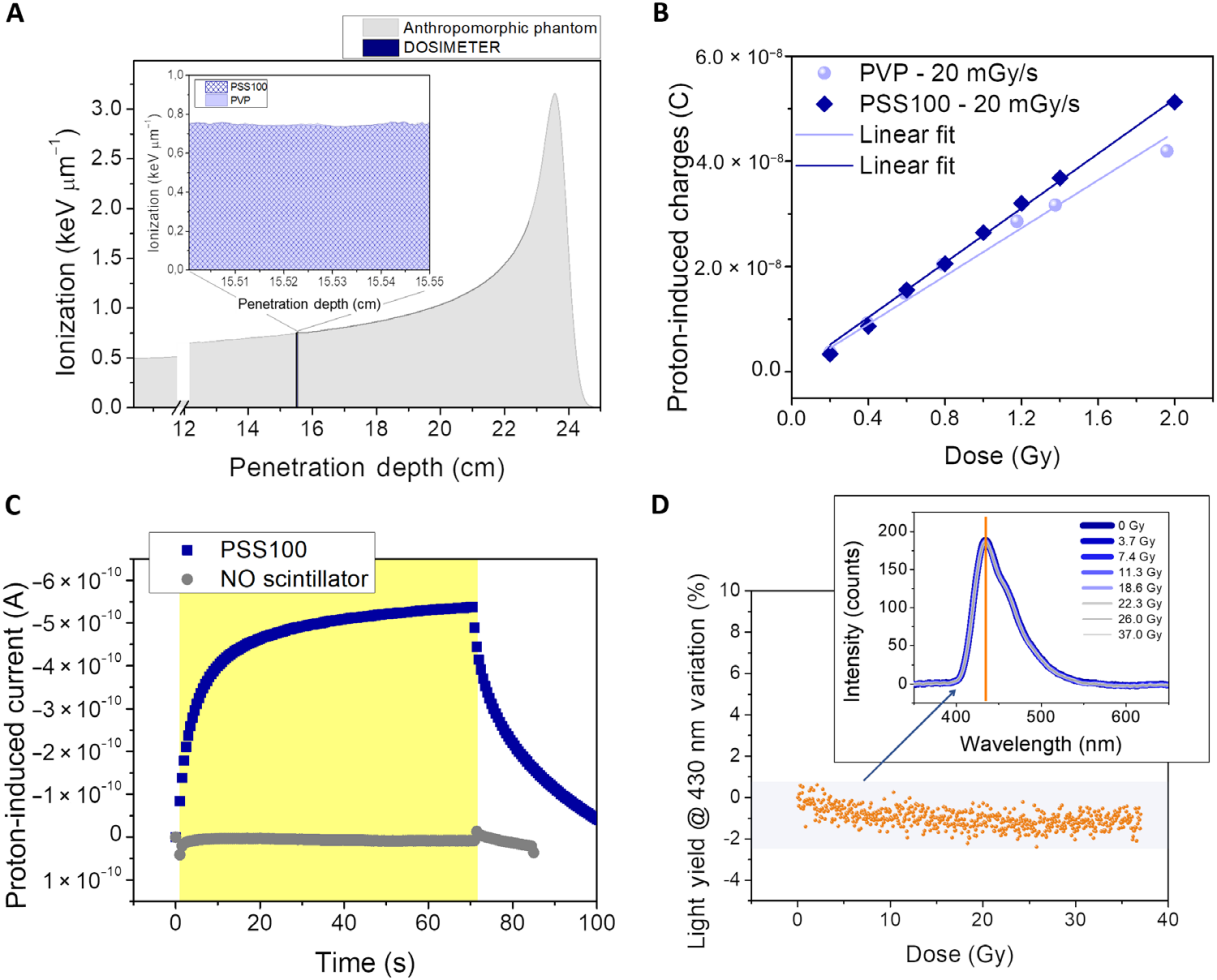
Polysiloxane scintillator characterization under high-energy proton beams. (**A**) Monte Carlo simulation (SRIM) of a 200-MeV proton beam ionization along human tissues. At 15.5 cm depth, a 500-μm–thick polysiloxane-based scintillator is placed. Inset: Zoom of the energy release within the PSS100 (blue texture) and PVP (light blue). (**B**) Proton-induced charges in an OPT coupled with a PSS100-based scintillator (blue squares) and with a PVP-based scintillator (light blue circles). (**C**) Dynamic response under 1.4 Gy (70-s exposure time, 20 mGy s^−1^) of a bare OPT (gray circles) and an OPT coupled with a PSS100-based scintillator (blue squares). (**D**) Ion beam–induced light measurement performed by irradiating a 20-mm–thick PSS100 scintillator with a 37-MeV proton beam (60 mGy s^−1^). Inset: Emission spectrum acquired every second (i.e., 3.7 Gy) up to 10 min of total irradiation (i.e., 37 Gy). Graph: Light yield variation at 430 nm. The degradation of the induced luminescence after 37 Gy is below 2%.

The very low radiation absorption fraction offered by organic thin films represents one of the greatest advantages of this technology for dosimetry application. Despite the low amount of energy deposited within the dosimeter, the detector allows to provide an effective induced signal thanks to the photoconductive gain and to the OPT amplification mechanism. The limited interaction between the primary proton beam and the device ensures low interference with the therapeutic radiation field, preventing its perturbation. This unique property opens the possibility of interposing the dosimeter between the radiation source and the patient during the irradiation to monitor the dose delivered in real time and in situ without interfering with the treatment plan, i.e., in transmission mode. Figure 3A reports the Monte Carlo simulation (SRIM) of the energy released by a 200-MeV proton beam along the anthropomorphic phantom. The blue line at 15.5 cm depth simulates the release of energy within the polysiloxane-based scintillators (both PVP and PSS100 are reported zoomed in the inset) centered in the prostate position. The 200-MeV proton beam releases 0.75 keV μm^−1^ (i.e., 375 keV in the whole scintillator), and from the reported spectrum, it is clear that, by interposing the dosimeter, we do not affect the Bragg peak position and the ionization of tissues beyond it. The two polysiloxane-based scintillators absorb the same amount of energy from the proton beam (inset in Fig. 3A), and when coupled with the OPT, they provide comparable output signals when irradiated at the same dose conditions (Fig. 3B).

With the aim to assess the full control of the here reported technology and to exclude any spurious signal coming from the integrated indirect detector, also considering the transparency of the scintillator to the ionizing radiation, we tested the device under the same irradiation conditions (70-s time of exposure, 20 mGy s^−1^, 1.4 Gy, *V*_DS_ = −1 V, *V*_GS_ = −4 V) with and without the coupled PSS100 scintillator. The signals collected under 200-MeV proton irradiation, reported in Fig. 3C, clearly show that the extra-charges generated within the semiconducting layer of the OPT are due only to the detection of the visible light emitted by the scintillator and not to primary protons passing through the scintillator and impinging onto the OPT.

Last, we tested the radiation hardness of the polysiloxane-based scintillator, under extreme conditions as for dose rate, total dose, and energy release, which largely exceeds the typical parameters used in clinical treatment. This approach is adopted aiming at the detection of even minor changes in scintillation performance due to eventual radiation damages. Thus, to test a radiation harsh environment and exceed the typical irradiation conditions occurring during the treatments, we irradiated a 20-mm–thick PSS100 using a 37-MeV proton beam (i.e., completely absorbed within the scintillator) at 60 mGy s^−1^. Every second, we acquired the ion beam–induced luminescence by coupling the lateral surface to an optical fiber, delivering the optical signal to the spectrometer (Ocean Optics) (see the inset in Fig. 3D). The plot in Fig. 3D reports the degradation of the light yield at 430 nm below 2% after 10 min of measurements and a total absorbed dose of 37 Gy, demonstrating an excellent stability and radiation tolerance of the scintillating material.

As for the radiation tolerance of PVP-MPS, we can rely on the results reported in our previous work (*21*), where both PSS100 and PVP-MPS (500 μm thick) underwent proton irradiation under similar conditions as for dose rate and energy loss per unit length. In that case, light collection through a power meter before and after irradiation demonstrated negligible changes up to total dose as high as 20 Gy for both the scintillator, showing their similarity in terms of radiation tolerance.

The radiation tolerance demonstrated by both the components of the indirect detector (i.e., OPT and scintillator) guarantees the stability of the detection response along the entire proton therapy irradiation session (i.e., typically 2 Gy per fraction).

**Fig. 4.**
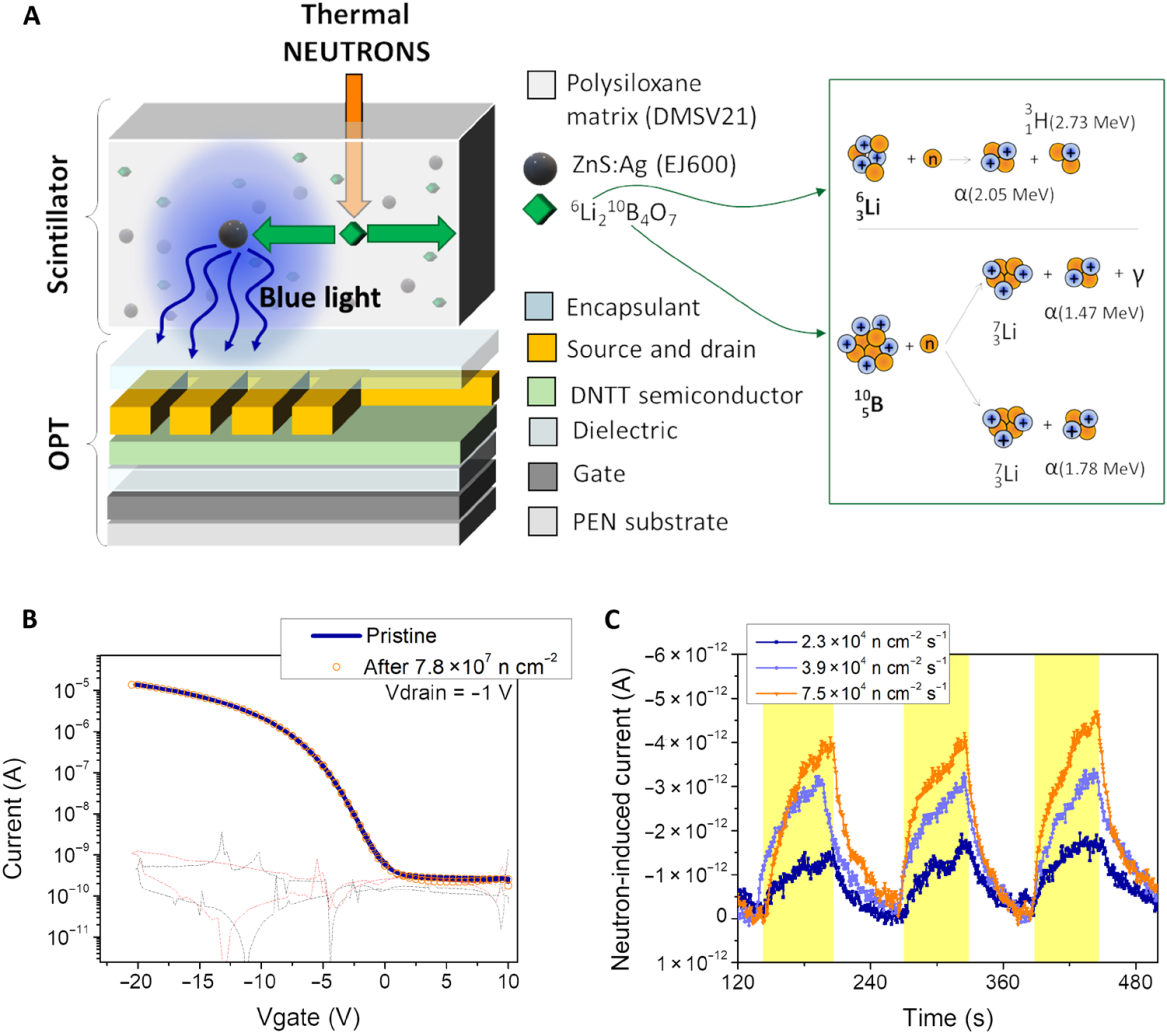
Flexible indirect detector for thermal neutrons. (**A**) Sketch of a full-organic indirect detector used for the detection of thermal neutrons. The polysiloxane scintillator (DMSV21 polymeric matrix) is functionalized with ZnS:Ag (EJ600) and ^6^Li_2_^10^B_4_O_7_ nanoparticles. The thermal neutrons interact with the LiBO nanoparticles following the nuclear reactions depicted on the right. The alpha particles produced are detected by ZnS:Ag, which emits blue light (λ = 450 nm). The light is detected by the OPT optically and mechanically coupled under the scintillator. The emission and absorption spectra of the scintillator and OPT are reported on the right, confirming the excellent optical coupling of the two systems. (**B**) Transfer curves of the detector before and after exposure under irradiation of 7.8 × 10^7^ total neutrons cm^−2^. (**C**) Dynamic response of a detector with a LiBO-based scintillator for increasing fluxes of thermal neutrons (2.3 × 10^4^, 3.9 × 10^4^, and 7.5 × 10^4^ n s^−1^ cm^−2^) irradiating the detector (time of exposure of 60 s highlighted in yellow). OPT working polarization: *V*_DS_ = −1 V, *V*_GS_ = −4 V.

To demonstrate the potential of the here discussed fully organic indirect detectors as personal dosimeters during different cancer radiation treatments, we coupled the same OPT with a differently functionalized dedicated polysiloxane scintillator. In particular, we tested a polysiloxane scintillator (DMSV21 polymeric matrix) functionalized with an inorganic part: ZnS:Ag (EJ600) and ^6^Li_2_^10^B_4_O_7_ nanoparticles as shown in the schematic of Fig. 4A. The addition of ^6^Li_2_^10^B_4_O_7_ nanoparticles allows to increase the interaction cross section with thermal neutrons by exploiting the nuclear reaction reported in Fig. 4A on the right. The secondary products (i.e., alpha particles, ^3^H, and ^7^Li) produced by these reactions interact with the EJ600, which emits light by scintillation (*26*, *27*). The visible photons are then detected by the OPT, generating an electrical output signal. The detection of thermal neutrons is a crucial task in some important medical fields such as the BNCT where, as for proton therapy, also in this case, some issues such as the effects of by-products of the primary beam and patient misalignment can cancel the selective aim of such treatments, exposing the patient to a huge radiation risk.

We tested our flexible, fully organic indirect detectors under the MUNES thermal neutron source at the Van de Graaff accelerator at the National Institute of Nuclear Physics–Legnaro Laboratory (INFN-LNL, CN accelerator, +15° beamline). This source exploits the ^9^Be(p,n)^9^B reaction, using a 5-MeV proton beam directed at a beryllium-vanadium target. The fast neutron spectrum provided by this reaction, centered around 1.2 MeV, is properly thermalized by a beam-shaping assembly (BSA) formed by heavy water, polytetrafluoroethylene (PFTE), and graphite. In addition, the detectors are placed behind a bismuth shielding window for the gamma-ray attenuation of the mixed radiation field. More details about the irradiation facility are reported in the literature (*28*, *29*) and in the Materials and Methods.

In Fig. 4B, the transfer characteristics of the OPT before and after the neutron irradiation with a flux of 7.8 × 10^7^ n cm^−2^ is reported. The overlap between the two curves indicates the excellent radiation hardness of the device.

Figure 4C shows the preliminary dynamic response of the detector acquired during irradiation with three subsequent 60-s irradiation cycles gradually increasing neutron beam flux ([2.3; 3.9; 7.5] × 10^4^ n s^−1^ cm^−2^). During the experiment, the sample has been biased using *V*_DS_ = −1 V, *V*_GS_ = −4 V, and the thermal neutron flux has been monitored using a calibrated detector assembly consisted on a 6-mm-diameter disk of a siloxane/^6^LiF (*26*) scintillator coupled to a Hamamatsu photomultiplier H6520 (operated at −700 V). The detector signals were acquired and processed online using a CAEN digitizer DT5725 controlled by the ABCD software (*27*).

The detecting response scales with the intensity of the thermal neutron beam even if, in this case, the linearity of the induced signal with the radiation intensity is poorer. We speculate that this main limitation is related to the low fluxes range used for this irradiation tests, which induce a detecting signal very low and close to the lowest limit of detection of the OPT [i.e., 3 nW cm^−2^ (*22*)]. This characterization represents only preliminary results showing the great potential of this detecting platform for the realization of a wearable and real-time dose monitoring tool to be used during BCNT where higher fluxes of epithermal neutrons are typically used (10^8^ to 10^9^ n cm^−2^ s^−1^).

## DISCUSSION

In this study, we present a fully organic, wearable, human tissue-equivalent dosimeter for real-time and in situ monitoring of radiation absorbed by patients during cancer treatments. The device, formed by an OPT and a polysiloxane-based scintillator, demonstrates excellent performance under 200-MeV proton irradiation under actual clinical conditions. By simulating prostate cancer treatment with a 200-MeV proton beam and by using an anthropomorphic phantom, we validate the device capability to accurately monitor the spatial distribution of the delivered dose. This novel and promising class of dosimeters offers several advantages, mainly (i) mechanical flexibility and large area scalability; (ii) human tissue equivalence, which eliminates the need for complex calibration procedures; (iii) thin film design, which allows for transmission operation mode without perturbing the radiation field or treatment plan; and (iv) excellent linear response for doses above 220 mGy, validating the device efficacy for real-time monitoring during treatments. The stable and reproducible response even after prolonged and intense radiation exposures is also noteworthy; (v) the ability to detect different types of ionizing radiation relevant for medical therapy (i.e., protons and thermal neutrons) through a proper and dedicated functionalization of the scintillator. On the other hand, despite all these potentialities, some improvements still need to be addressed such as (i) assessing the reproducibility on large areas, (ii) boosting spatial and temporal resolution, and (iii) challenging in designing and integration of multichannel readout electronics compatible with this wearable and portable detecting technology.

Overall, this fully organic indirect detection platform shows a great potential as a tool to enhance the precision and quality of cancer treatments by providing real-time and in situ accurate dose monitoring. The prospect to integrate this technology into actual clinical settings could lead to substantial improvements in radiation therapy outcomes and patients’ safety.

## MATERIALS AND METHODS

### Scintillator fabrication

The thin PSS100 and PVP-MPS scintillators have been produced as described previously (*21*). Briefly, the precursor resin, either polymethylphenylsiloxane vinyl terminated for PSS100 or poly(vinylphenyl)-*co*-(methylphenyl) vinyl terminate for PVP-MPS (GELEST), has been added with suitable fluorophores [2,5-diphenyloxazole (1% wt) and Lumogen Violet, BASF (0.02% wt)]. After complete dissolution through mixing, the cross-linker, hydride terminated siloxane resin, and Pt-based catalyst have been added. Then, the resin was cast onto a glass plate previously coated with a release agent and allowed to cross-link at 60°C for 18 hours. Last, the thin siloxane layer with a thickness of 0.5 mm was removed from the glass by simple immersion in warm deionized water.

The thermal neutron scintillator has been prepared according to the procedure described in a previous work (*27*). For the experiment herein described, the nanocomposite was 0.5 mm thick and contained the powder mixture ZnS:Ag/LiBO (2:1) (40%, v/v)/polydimethylsiloxane (PDMS), where LiBO stands for ^6^Li_2_^10^B_4_O_7_ in the form of nanocrystals prepared by direct synthesis, whereas PDMS is a commercial addition curable PDMS resin (DMS-V21; Gelest Inc.).

### OPT fabrication

The OPT devices have been fabricated in a cleanroom environment at low temperatures (*22*). The devices are made with staggered bottom-gate, top-contact configuration, which was demonstrated to improve the performance of the transistor by reducing the contact resistance compared to other configurations (*30*, *31*). The used flexible freestanding substrate is a 100-μm–thick PEN foil upon which the Al gate electrode (70 nm thick) was formed by shadow mask patterned evaporation. The dielectric layer (650 nm thick) is composed of the fluoropolymer Cytop. The semiconductor (DNTT) was then evaporated in high vacuum conditions with a deposition rate of 0.01 nm/s, keeping the substrate at room temperature. The source and drain interdigitated electrodes (Au, 30 nm thick) and the connections (Al, 50 nm thick) have been evaporated in HV and patterned by shadow masks. Last, the passivation layer is composed of a double stack organic film: 400 nm of parylene deposited in vacuum at room temperature in a solvent-free process and 240 nm of Cytop. A complete optoelectronic characterization of the OPTs has been performed, demonstrating top-notch performances with excellent electrical and mechanical reliability as well (*22*), high sensitivity for very small optical signals (*32*), and the lowest electronic noise measured for organic devices so far (*33*).

### Anthropomorphic phantom

The anthropomorphic phantom was produced by means of 3D printing, based on the actual computed tomography (CT) of a patient. Therefore, it closely resembles a real human anatomy. The materials used were PA12 and chalk powder.

### Monte Carlo simulation

The biological dose released (RBE 1.1) was calculated for a prostate cancer representative treatment plan with two opposing proton fields, obtained by means of the TPS Raystation (Raysearch Laboratories) with the Monte Carlo dose computation engine. The proton beam energy was in the range (162 to 197 MeV).

### Proton irradiation

The dosimeter was characterized at the experimental room of the Trento Proton therapy Center (APSS, Azienda Provinciale per i Servizi Sanitari) under clinical-like conditions. A 200-MeV proton beam was delivered, and the detector was placed in a dedicated housing inside the anthropomorphic phantom, mimicking the position of the prostate (i.e., centered in the primary beam). The beam inside the phantom was attenuated to 118 MeV because of the release of energy in the 15.5-cm human tissue traversed before impinging onto the device. We irradiated the sample varying the exposure time in the range [10 to 100] s. The dose released by the proton beam was calibrated using a commercial dosimeter (MARKUS) and the ionization chamber. The dose rate was set at 20 mGy s^−1^. During the irradiation, the OPT was electrically connected, biased in dc (*V*_DS_ = −1 V, *V*_GS_ = −4 V) and the *I*_DS_ current was monitored in real time using a benchtop source meter. In addition, the device was tested under the same conditions, being read out by a custom-designed transimpedance amplifier featuring a gain of 50 × 10^6^ V A^−1^ and a bandwidth of 300 kHz, whose output was recorded using a 24-bit data logger. This alternative setup offers a more compact and efficient solution compared to commercial source measurement units and potentially more practical for real medical applications.

### Neutron irradiation

The measurements were conducted at the MUNES thermal neutron source, located along the +15° beamline of the CN accelerator at the LNL. This source generates neutrons through the ^9^Be(p,n)^9^B reaction, using a 5-MeV proton beam directed at a beryllium-vanadium target. The fast neutron spectrum, centered around 1.2 MeV, is tailored to the thermal energy range using a large BSA composed of 4014 kg of graphite, 88.5 kg of heavy water, 53 kg of PFTE, and 24 kg of bismuth. The BSA, occupying a volume of about 3 m^3^, facilitates the production of thermal neutrons, which are extracted through a bismuth exit channel with a surface area of 10 cm by 10 cm.
