# Supplementary material for: A wearable tool for real-time dose monitoring during cancer radiation therapies
Source: Sci Adv. 2025 Apr 25;11(17):eadt7633. doi: 10.1126/sciadv.adt7633 (PMC12024650; doi:10.1126/sciadv.adt7633)
Supplement: Supplementary file 1 — Table S1 Figs. S1 to S5 [file sciadv.adt7633_sm.pdf]

Science Advances

Supplementary Materials for

A wearable tool for real-time dose monitoring during cancer radiation therapies

Ilaria Fratelli *et al.*

Corresponding author: Laura Basiricò, laura.basirico2@unibo.it

Sci. Adv. **11**, eadt7633 (2025)
DOI: 10.1126/sciadv.adt7633

This PDF file includes:

Table S1
Figs. S1 to S5

Material	Zeff	Electron density (cm^{-3})
PSS-100	9.02	4.32E+22
PVP-MPS	8.77	3.44E+22
ICRU, soft tissue	7.26	9.87E+22
ICRU, Muscle Striated	7.46	7.48E+22
Water liq.	7.42	2.48E+23
PMMA	6.47	4.59E+22

Table S1. Effective Atomic Number and Electron Density of polysiloxane based scintillators. Effective Atomic Number (Zeff) and Electron Density of both polysiloxanes used as matrices, calculated starting from the stoichiometric composition and mass density. Other typical values recognized as tissue-equivalent standard materials (ICRU-soft-tissue, ICRU-Muscle-striated, Water, PMMA) are reported.

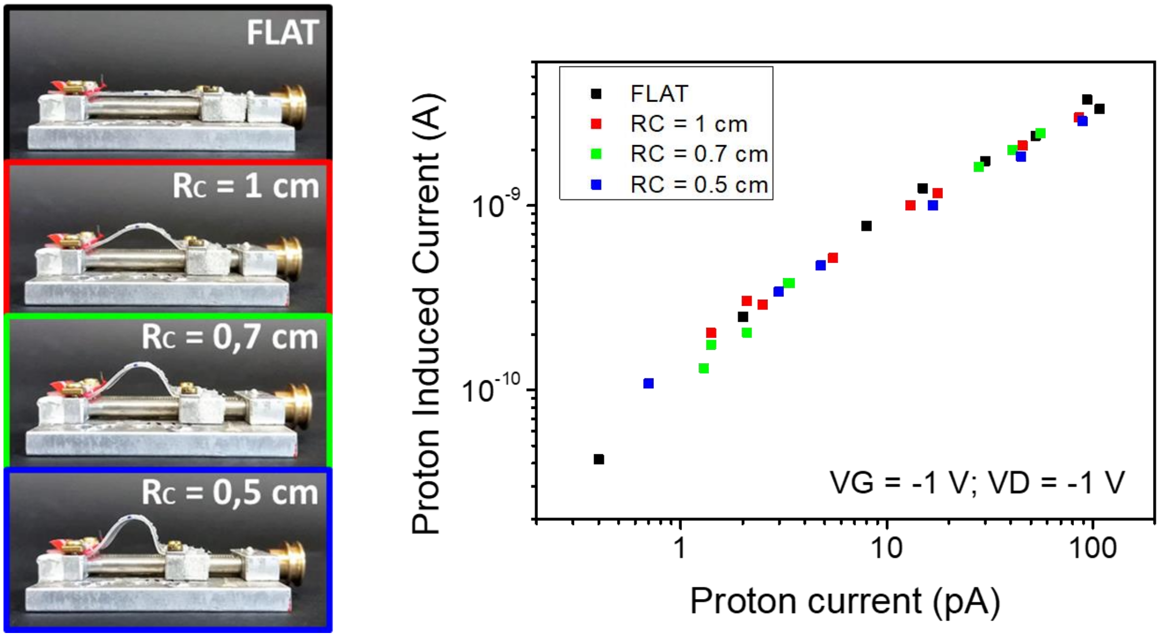

Fig. S1. Mechanical flexibility of the detecting system under proton irradiation.

Mechanical flexibility test. Proton induced current at different 5 MeV proton fluxes (proton current in the range [0.1 - 100] pA) acquired by the organic detector in the flat condition (black) and while it was kept bent at different curvature radii (1 cm (red), 0.7 cm (green), 0.5 cm (blue)). The induced signal under radiation has been acquired when the sample was kept bent and the comparable responses demonstrate the excellent mechanical flexibility of the detector.

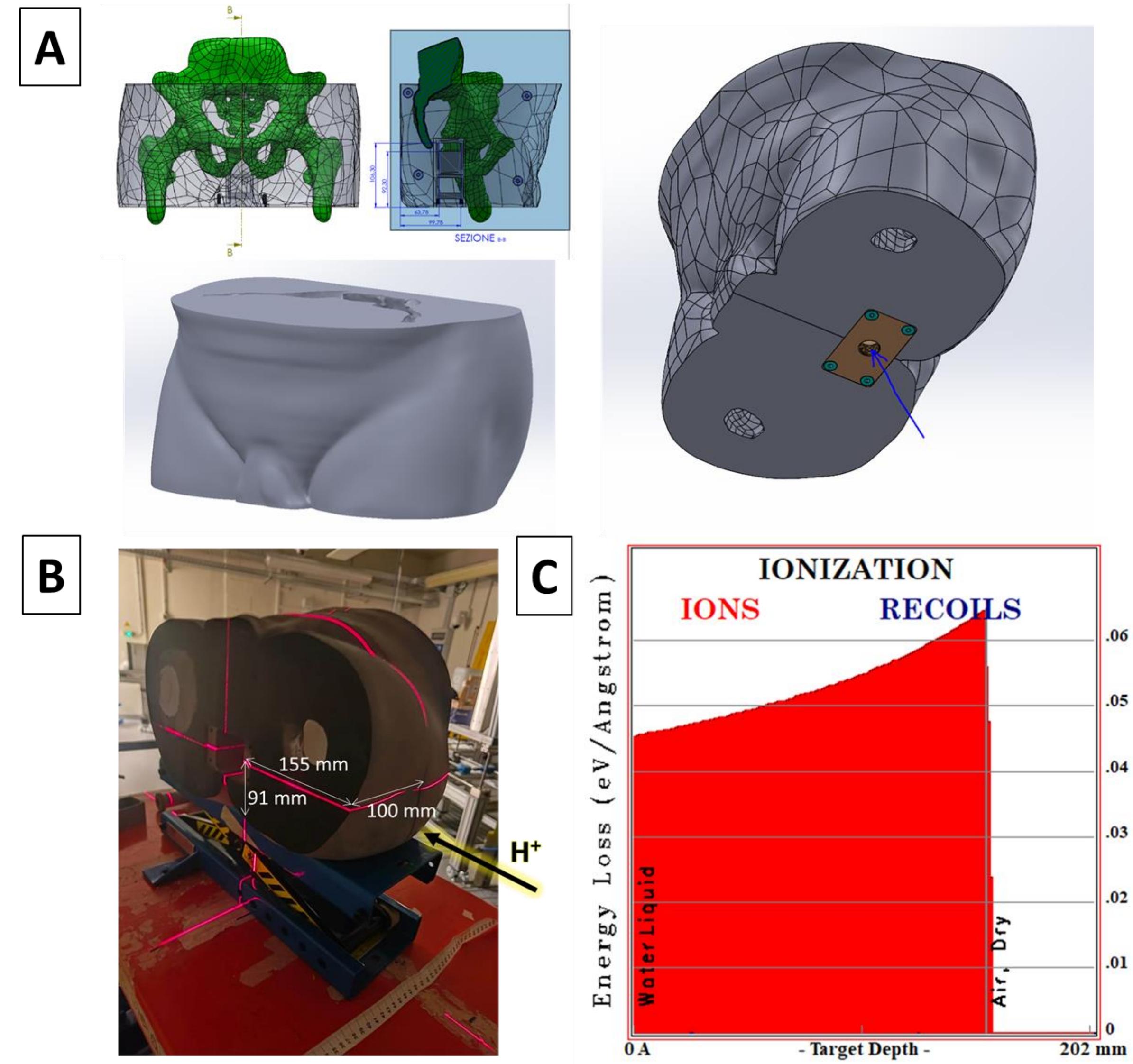

Fig. S2. Anthropomorphic Phantom for in-situ characterization.

A) Anthropomorphic phantom. The phantom is made by human tissue equivalent materials realized by 3D printing. The detector is placed inside a box at the rectum position and indicated by the blue arrow. (B) During the prostate cancer treatment, the proton beam enters from the side of the body and it travels 15.5 cm before reaching the target point. (C) Monte Carlo simulation about the energy released by 200 MeV proton passing 15.5 cm of human tissue equivalent material before impinging onto the detector. The beam energy at the detector position is 118 MeV.

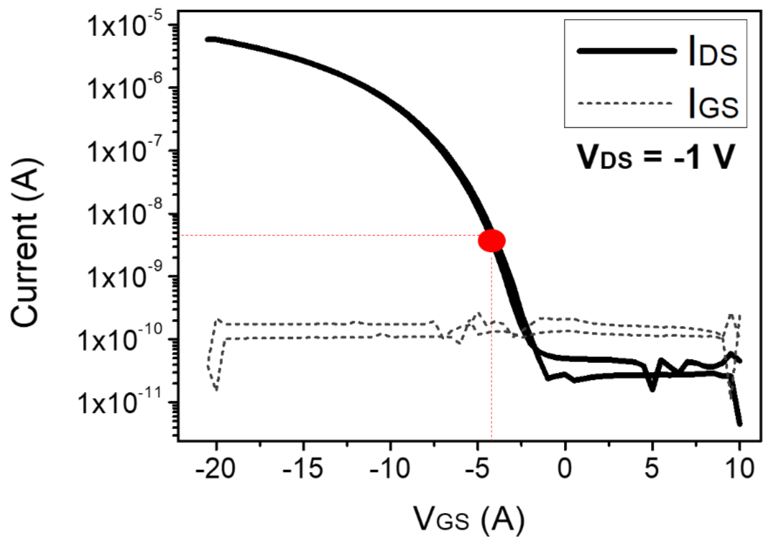

Fig. S3. Organic Photo-Transistor working Point.

OPT transfer characteristic in linear regime (V_{DS} = -1 V). The red dot indicates the working point of the detector (V_{GS} = -4 V).

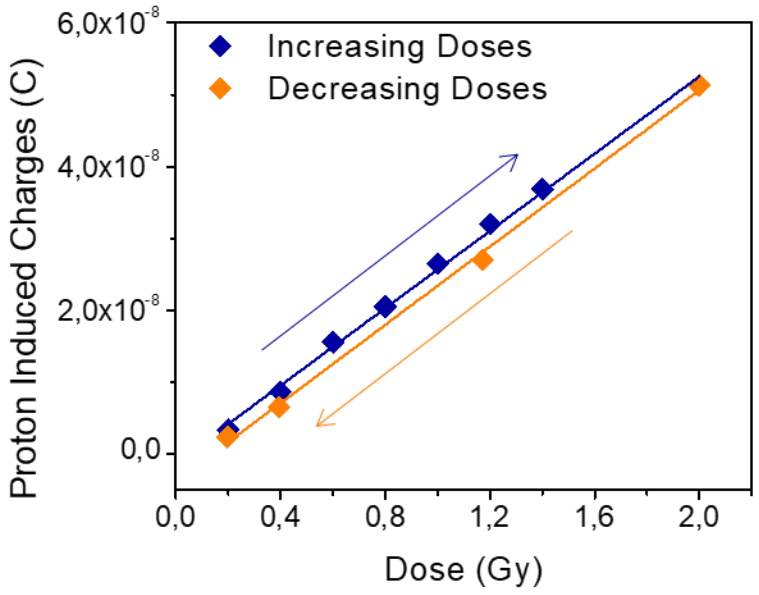

Fig. S4. Repeatability of the proton detection response.

Proton induced charges at different total doses. The blue spheres are for the increasing doses while the orange squares are for the decreasing doses. The overlapping between the curves indicates the reproducibility of the detecting response provided by the detector.

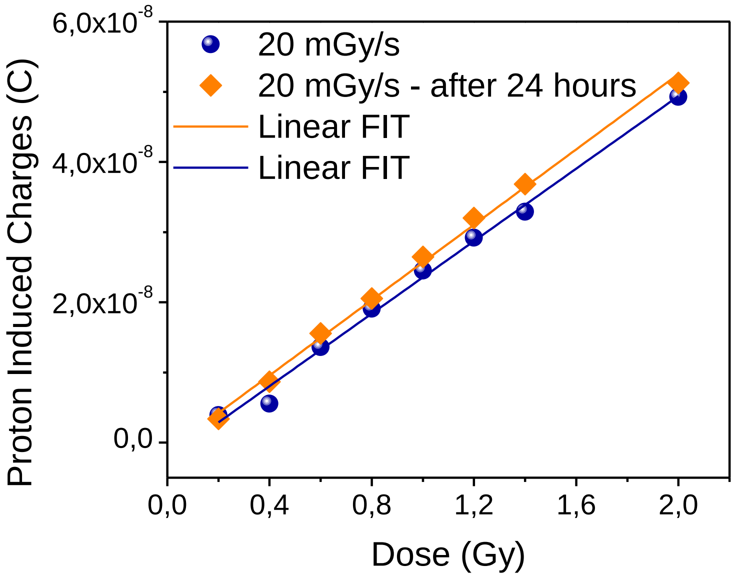

Fig S5. Stability of the Proton Detection Response.

Proton induced charges at different total doses. The blue spheres are for the initial exposure while the orange squares are for the measurements performed after 24 hours. The overlapping between the curves indicates the stability of the detecting response provided by the detector.
